# Effects of catalase on chloroplast arrangement in *Opuntia streptacantha* chlorenchyma cells under salt stress

**DOI:** 10.1038/s41598-017-08744-x

**Published:** 2017-08-17

**Authors:** Diana Marcela Arias-Moreno, Juan Francisco Jiménez-Bremont, Israel Maruri-López, Pablo Delgado-Sánchez

**Affiliations:** 1 Laboratorio de Biotecnología, Facultad de Agronomía y Veterinaria, Universidad Autónoma de San Luis Potosí, Soledad de Graciano Sánchez, San Luis Potosí, Mexico; 2 Laboratorio de Biología Molecular de Hongos y Plantas, División de Biología Molecular, Instituto Potosino de Investigación Científica y Tecnológica A.C, San Luis Potosí, Mexico

## Abstract

In arid and semiarid regions, low precipitation rates lead to soil salinity problems, which may limit plant establishment, growth, and survival. Herein, we investigated the NaCl stress effect on chlorophyll fluorescence, photosynthetic-pigments, movement and chloroplasts ultrastructure in chlorenchyma cells of *Opuntia streptacantha* cladodes. Cladodes segments were exposed to salt stress at 0, 100, 200, and 300 mM NaCl for 8, 16, and 24 h. The results showed that salt stress reduced chlorophyll content, *F*_*v*_/*F*_*m*_, ΦPSII, and *qP* values. Under the highest salt stress treatments, the chloroplasts were densely clumped toward the cell center and thylakoid membranes were notably affected. We analyzed the effect of exogenous catalase in salt-stressed cladode segments during 8, 16, and 24 h. The catalase application to salt-stressed cladodes counteracted the NaCl adverse effects, increasing the chlorophyll fluorescence parameters, photosynthetic-pigments, and avoided chloroplast clustering. Our results indicate that salt stress triggered the chloroplast clumping and affected the photosynthesis in *O*. *streptacantha* chlorenchyma cells. The exogenous catalase reverted the H_2_O_2_ accumulation and clustering of chloroplast, which led to an improvement of the photosynthetic efficiency. These data suggest that H_2_O_2_ detoxification by catalase is important to protect the chloroplast, thus conserving the photosynthetic activity in *O*. *streptacantha* under stress.

## Introduction

To ensure their survival, plants have evolved to optimize the capture of energy and nutrients^1^. Previous studies have shown that organelles, such as mitochondria, peroxisome, and chloroplast are constantly moving within the cells taking specific positions to maximize their metabolic activities during changing environmental conditions^2–4^. In particular, the chloroplast movement in response to light (photo-relocation) is essential for the survival of plants under extreme light conditions^5, 6^. Additionally, chloroplast movement has a protective role in response to other abiotic stresses, such as drought and low temperatures^7–9^. Chloroplast movement has been extensively studied in the model plant *Arabidopsis thaliana*, operating with C_3_ type photosynthesis^5^. However, only a few studies have been undertaken about chloroplast movement in C_4_ and CAM (Crassulacean Acid Metabolism) plants^7, 9–11^. Previously, our research group reported that the *Opuntia streptacantha* chloroplasts were grouped together within the cells under combined light and water stress, which probably maintain its photosynthetic process active in CAM plants^9^.

Particularly, the high plasticity in the CAM photosynthetic pathway is one of the most successful physiological strategies for plant acclimation and adaptation to water shortage^12^. The water loss is minimized because during the daytime CAM plants photosynthesize with closed stomata, using the CO_2_ that was stored in the vacuole in form of organic acids, mainly malic acid, during night^12^. Thus, CAM plants are able to survive under extreme abiotic stress conditions, predominantly scarcity of water and extreme temperatures, in contrast to C_3_ and C_4_ plants^13^.

*Opuntia streptacantha* is an endemic cactus from Mexico, which is distributed along the southern Chihuahuan Desert^14^. It performs a CAM type photosynthesis, which permits a successful establishment of intracellular levels of the plant in arid and semiarid regions of many countries^12, 14, 15^. Despite the ability of CAM plants to overcome water stress, not all CAM plants are successful to cope salinity^12^.

It is known that salinity affects the photosynthesis process in plants^16^. An early effect of salt stress is stomatal closure, which leads to a restriction of CO_2_ diffusion into the cells and chloroplasts^17^. Subsequently, the increase of reactive oxygen species (ROS) levels, can seriously affect the plant’s photosynthetic machinery^18, 19^. However, ROS are also important signaling molecules involved in the control of plant growth, development, photosynthetic functions, and responses to biotic and abiotic stress^19–21^. In particular, the hydrogen peroxide (H_2_O_2_) is the most significant non-radical ROS^22^. H_2_O_2_ is produced predominantly during the photosynthesis and photorespiration process in the apoplast, chloroplast, peroxisome, and mitochondria^20, 23^. Plants possess efficient scavenging systems for ROS, which protect them from destructive oxidative reactions. The principal H_2_O_2_ scavenging enzyme in plants is catalase (CAT), which directly converts H_2_O_2_ into H_2_O and O_2_^24^. Likewise, ascorbate peroxidase (APX) and glutathione peroxidase (GPX) are important enzymes essentials for the elimination of H_2_O_2_ levels in the cell^25^. The balance between ROS generation and ROS-scavenging during exposure to stressful environments is essential to regulate the mechanisms of ROS signaling in plants^26, 27^.

The negative effects on growth and CO_2_ uptake in *Opuntia* plants under salt stress have been previously reported^28–30^; however, little information about other physiological data related to salinity has been provided. In this study, our main objective was to examine the effect of NaCl treatments on chloroplast movement in chlorenchyma cells of *O*. *streptacantha*. Through optical microscopy, we observed the chloroplast arrangement under different treatments with NaCl (0, 100, 200, and 300 mM). Additionally, we measured diverse physiological parameters, such as the chlorophyll fluorescence, photosynthetic pigments content, and ultrastructure of chloroplasts to determine the effect caused by NaCl treatments. We found that the H_2_O_2_ accumulation, clustering of the chloroplasts, and a decline in photosynthetic activity were consequences of salinity in *O*. *streptacantha*. Finally, exogenous application of CAT enzyme was used to counteract the effects produced by salt treatment. Our data suggest that H_2_O_2_ accumulation is relevant for chloroplast clustering under salinity in *O*. *streptacantha*.

## Results

### The photosynthetic capacity of *O*. *streptacantha* cladodes decreases under salt treatments

**Figure 1:**
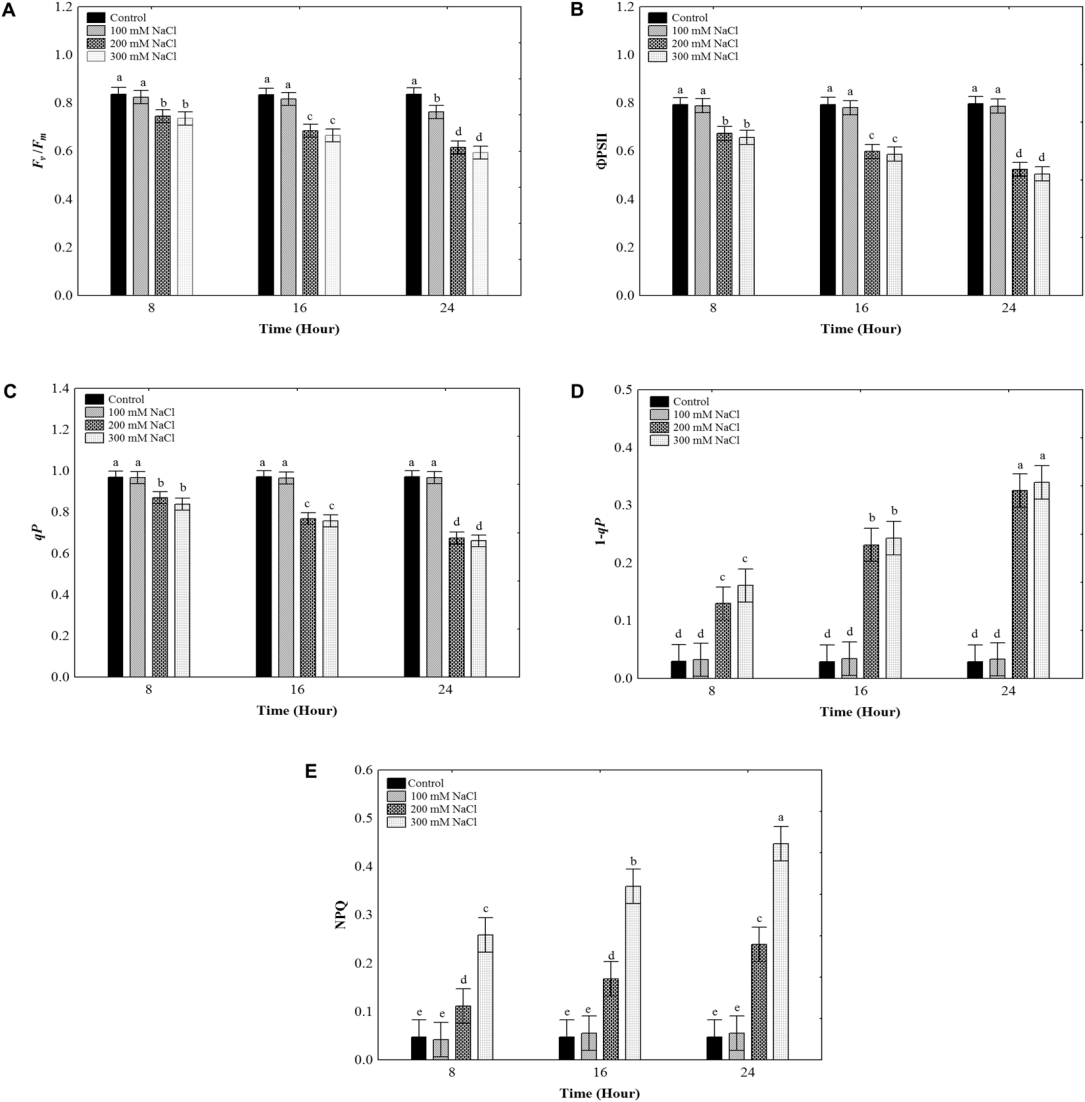
Chlorophyll fluorescence parameters in *Opuntia streptacantha* cladodes under salt treatments. (**A**) The maximum quantum yield of PSII (*F*_*V*_/*F*_*m*_). (**B**) The effective photochemical quantum yield of PSII (ΦPSII). (**C**) The photochemical quenching (*qP*). (**D**) The excitation pressure (1–*qP*). (**E**) The non-photochemical fluorescence quenching (NPQ). Values are means and bars indicate ± SD, (*n* = 9). Different letters indicate significant difference between treatments and time (hours) according to Duncan’s multiple range tests at *P* < 0.05.

We performed chlorophyll fluorescence measurements in *O*. *streptacantha* to evaluate the effect of salinity on photosynthesis, by using cladode segments exposed to salt treatments at 0, 100, 200, and 300 mM NaCl for 8, 16, and 24 h. The values of the maximum quantum yield of photosystem II (*F*_v_/*F*_m_), the effective photochemical quantum yield of PSII (ΦPSII), and the photochemical quenching (*qP*) showed a decreasing trend related to NaCl concentration and treatment duration (Fig. 1A–C). The excitation pressure (1-*qP*) and the non-photochemical fluorescence quenching (NPQ) values were significantly increased (Fig. 1D and E). At 100 mM NaCl for 8, 16, and 24 h, the values of *F*_v_/*F*_m_, ΦPSII, *qP*, 1-*qP*, and NPQ did not give statistically significant differences, whereas, in the treatment with the highest salt concentrations, the *F*_v_/*F*_m_, ΦPSII, and *qP* values were significantly reduced at the three time periods analyzed compared to the control. Meanwhile, the 1-*qP* and NPQ values were significantly increased. We also found that the photosynthetic electron transport rate (ETR) was significantly reduced at 200 and 300 mM NaCl compared to the control (Supplementary Fig. S1). These results showed that the NaCl concentration and exposure time affected negatively the photosynthetic capacity of *O*. *streptacantha* cladodes.

### Salt treatments effect on photosynthetic pigments content in *O*. *streptacantha* cladodes

**Figure 2:**
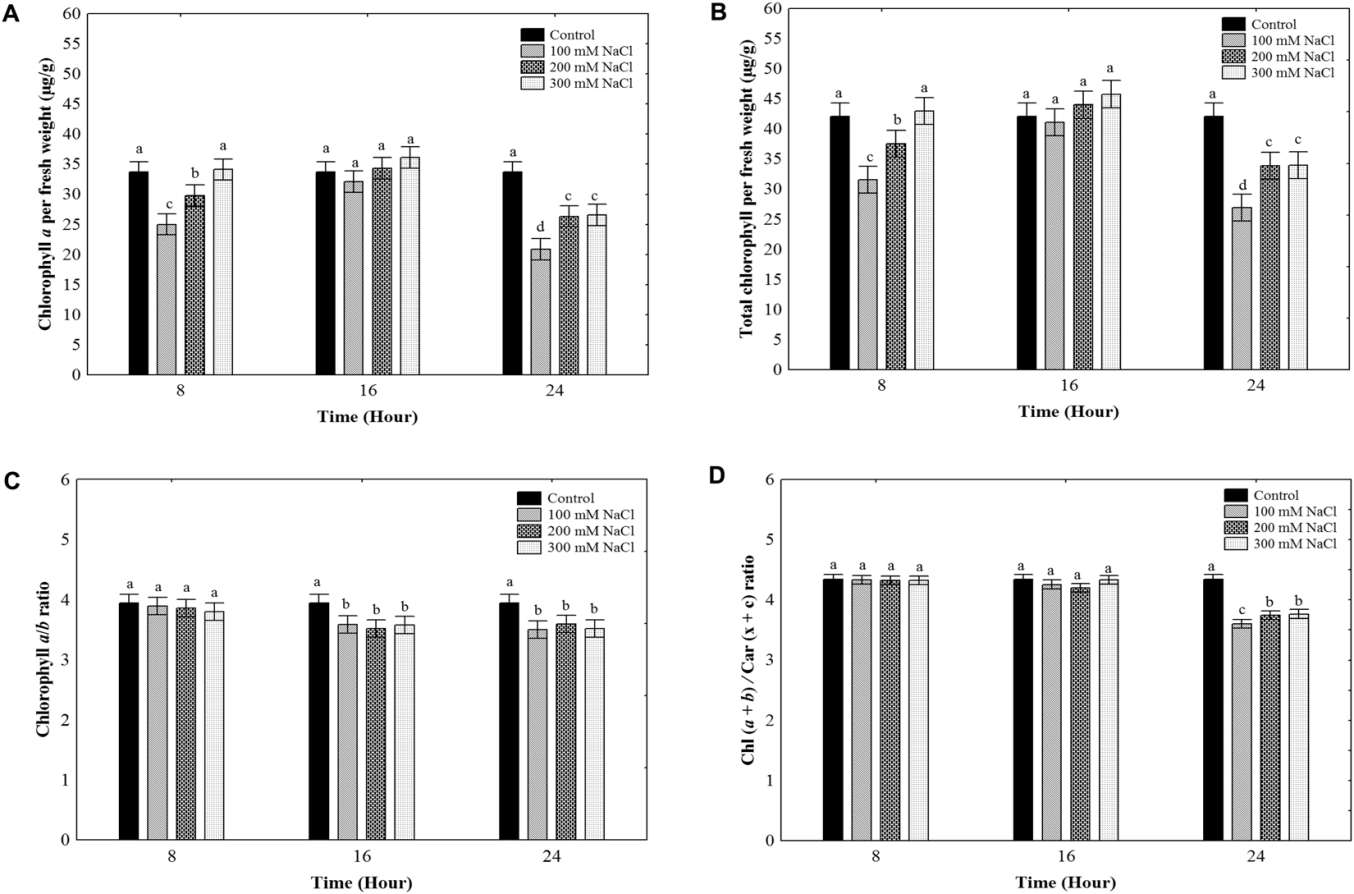
Chlorophyll and carotenoids content in *Opuntia streptacantha* cladodes under salt treatments. (**A**) Chlorophyll *a*. (**B**) Total chlorophyll (*a* + *b*). (**C**) Chlorophyll *a*/*b* ratio. (**D**) Chl (*a* + *b*)/Car (x + c) ratio. Values are means and bars indicate ± SD, (*n* = 9). Different letters indicate significant difference between treatments and time (hours) according to Duncan’s multiple range tests at *P* < 0.05.

In order to determine the effect of salt stress on the photosynthetic pigments content in *O*. *streptacantha*, we quantified the chlorophyll *a*, *b*, total (*a* + *b*), and carotenoids (x + c) content in cladodes segments exposed to salt treatments at 0, 100, 200, and 300 mM NaCl during 8, 16, and 24 h. We observed that the chlorophyll *a*, and total (*a* + *b*) contents tends to decline along with the exposure time to NaCl concentrations (Fig. 2). At 8 h of salt treatment, were observed a decrease in chlorophyll *a*, and total (*a* + *b*) levels at 100 and 200 mM NaCl concentrations compared to the control. However, no changes were observed in the Chl *a*, and total (*a* + *b*) levels at 16 h under the salt treatments evaluated. After 24 h of the salt treatments, we found a decrement of chlorophyll *a*, and total (*a* + *b*) levels in all the salt concentrations tested (Fig. 2A and B). On the other hand, we did not observe a significant difference in the ratio of Chl *a* to Chl *b* (Chl *a*/*b*) at 8 h of salt treatments. Meanwhile, the Chl *a*/*b* ratio was significantly reduced at 16 and 24 h for all the salt treatments (Fig. 2C). The ratio of Chl *a* and Chl *b* to total carotenoids (*a* + *b*)/(x + c) was significantly reduced only at 24 h of salt treatments (Fig. 2D). Therefore, salt treatments negatively affected the chlorophyll and carotenoids content in *O*. *streptacantha* cladodes.

### Salt treatments triggers chloroplasts clumping in *O*. *streptacantha* chlorenchyma cells

**Figure 3:**
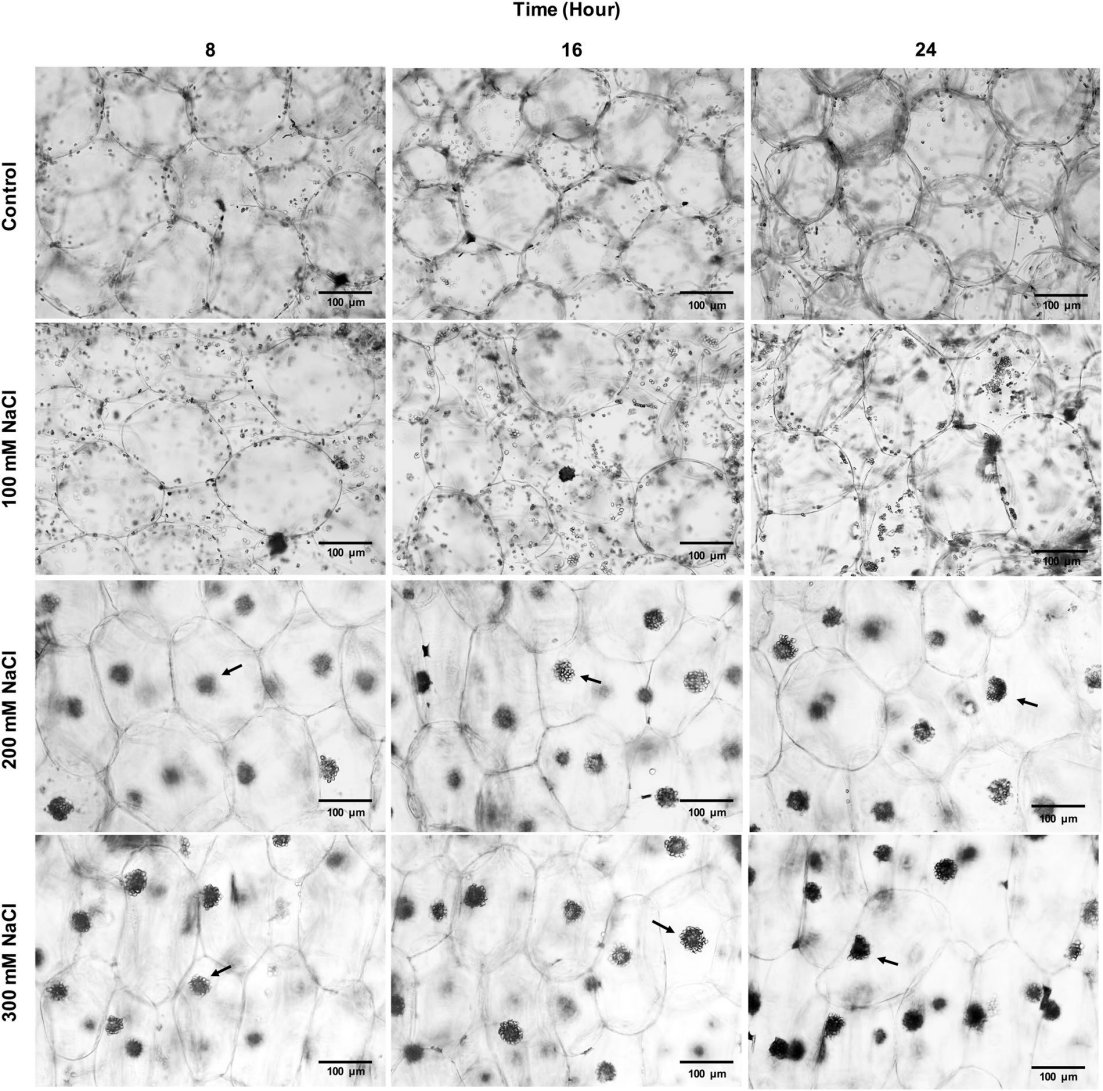
The chloroplast arrangement in chlorenchyma cells from *Opuntia streptacantha* under salt treatments. Representative images of *O*. *streptacantha* cells and their chloroplast are shown in each panel. Cladode segments were cut, and after they were incubated in 0, 100, 200, and 300 mM NaCl under continuous white light (300 µmol m^−2^ s^−1^) for 8, 16, and 24 h. Transverse sections from cladode were observed with a light microscope. The black arrowheads indicate chloroplast clusters. Scale bar corresponds to 100 μm.

In order to analyze the chloroplast arrangement in response to salt treatments, *O*. *streptacantha* cladode segments were incubated with 0, 100, 200, and 300 mM NaCl under continuous light conditions for 8, 16, and 24 h (Fig. 3). Under the control conditions, the chloroplasts were always dispersed in the cytosol of the cells. However, we observed chloroplast clumping in chlorenchyma cells when the salt concentration was increased. At 100 mM NaCl for 24 h the chloroplasts were redistributed. Furthermore, it was observed that in some cells chloroplasts began to cluster. Additionally, we detected aggregation of chloroplasts at 200 and 300 mM NaCl during the period analyzed. We also observed that NaCl-induced chloroplast clumping at 200 mM for 8 h was reversible when the salt stressed cladodes segments were washed and then incubated in distilled water (Supplementary Fig. S2). These results evidence that salt treatment triggers chloroplasts clumping in *O*. *streptacantha* chlorenchyma cells.

### Salt treatment induced ultrastructural changes in thylakoid membranes of *O*. *streptacantha* chlorenchyma cells

**Figure 4:**
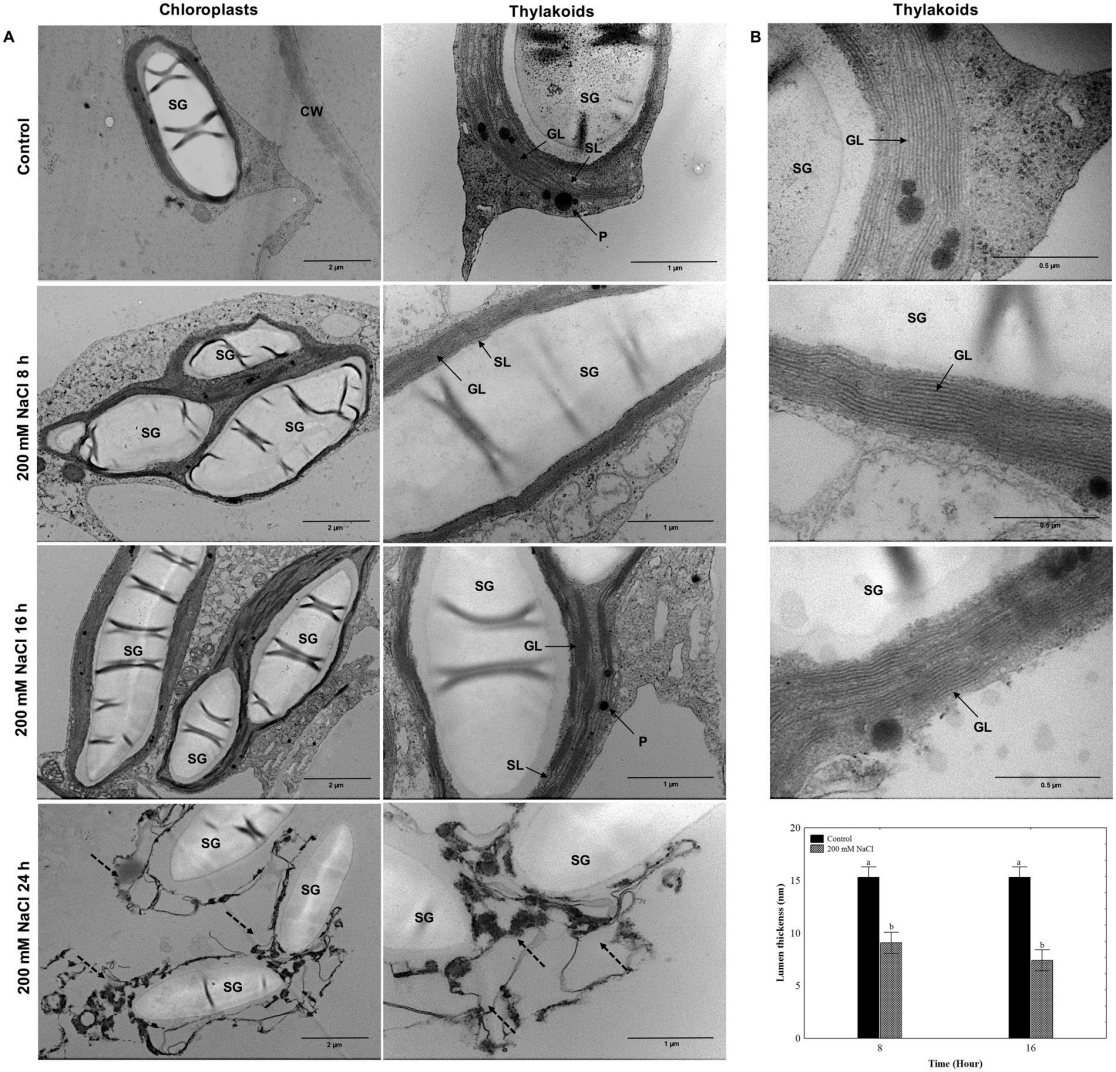
Ultrastructure of chloroplasts and thylakoid membranes of *Opuntia streptacantha* chlorenchyma cells under salt treatment. Representative TEM images of *O*. *streptacantha* chloroplasts are shown in each panel. (**A**) Cladode segments from *O*. *streptacantha* were incubated in 200 mM NaCl at 8, 16, and 24 h under white continuous light (300 µmol m^−2^ s^−1^). The left panel displays individual chloroplast micrographs; the scale bar corresponds to 2 μm. In the right panel thylakoid membranes are shown; the scale bar corresponds to 1 μm. The black arrow dotted indicates the fragmentation of thylakoid membranes. (**B**) Ultrastructural analysis of thylakoid membranes of *O*. *streptacantha* chloroplasts under 200 mM NaCl at 8 and 16 h. The scale bar corresponds to 0.5 μm. The graph shows analysis of lumen thickness of thylakoid grana membranes within intact chloroplasts. Different letters indicate significant difference between treatments and time (hours) according to Duncan’s multiple range tests at *P* < 0.05. SL, stroma lamella; GL, grana lamellae; SG, starch grain; P, plastoglobule; CW, cell wall.

To analyze if the ultrastructure of chloroplasts gets affected by exposure to salt treatment, cladode segments of *O*. *streptacantha* were incubated with 200 mM NaCl during 8, 16, and 24 h. Subsequently, the chloroplast membranes were analyzed by a transmission electron microscope (TEM). At the ultra-structural level, chloroplast distortion was observed in the cladode segments exposed to salt treatment for 8, 16, and 24 h compared to the control (Fig. 4A). Initially, we observed a stacking in the thylakoid membranes at 8 and 16  while at 24 h, a distortion of chloroplasts was highly visible where the thylakoid membranes were fragmented. These results show that the thylakoid membranes were notably affected by the salt treatment. In order to confirm the stacking of thylakoid membranes, measurements of lumen thickness were made on grana lamellae (GL) in separate micrographs (Fig. 4B). Electron microscopy data showed that the width of the thylakoid lumen was significantly diminished by the exposure to 200 mM NaCl during 8 and 16 h compared to the control. The lumen thickness at 24 h under salinity was not examined because the thylakoid membranes were severely distorted. These results indicate that salt treatment induced thylakoid membranes stacking in *O*. *streptacantha*.

### Catalase treatment avoid the clustering of chloroplasts under salt treatment

**Figure 5:**
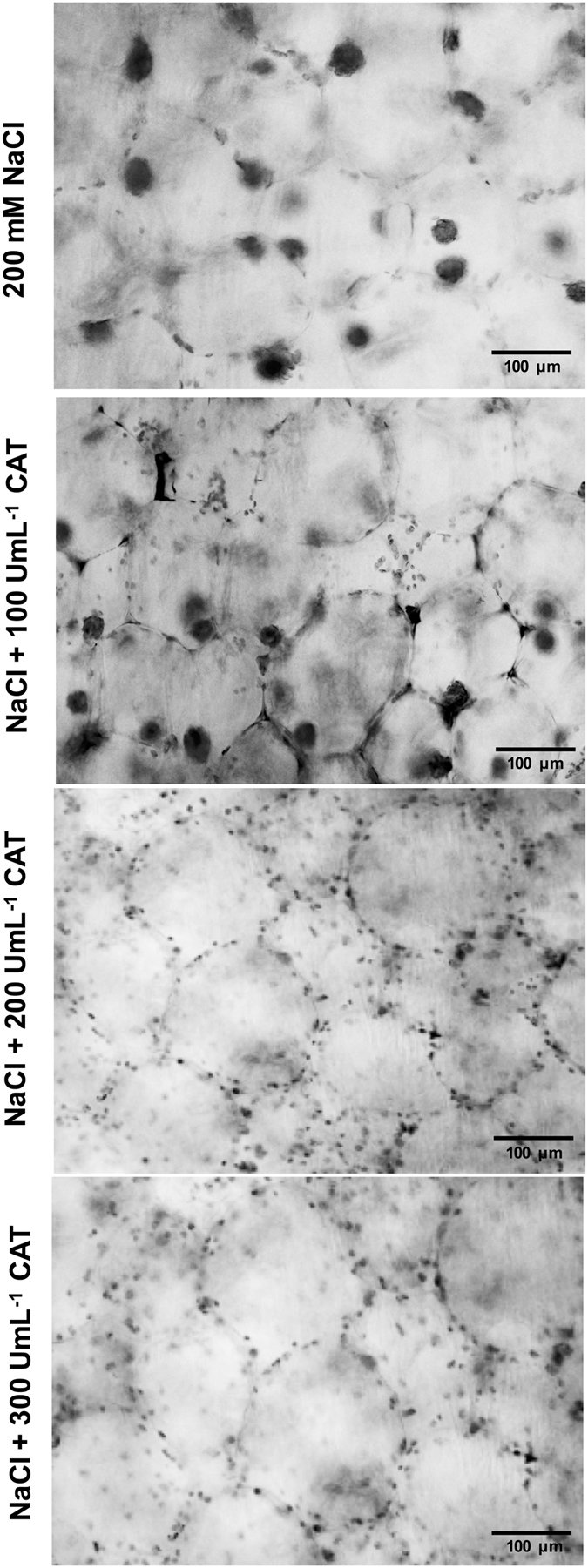
Chloroplast arrangement in chlorenchyma cells from *Opuntia streptacantha* under NaCl treatment supplemented with CAT. Representative images of *O*. *streptacantha* cells and their chloroplasts are shown in each panel. The cladode segments from *O*. *streptacantha* were incubated in 200 mM NaCl, 200 mM NaCl + 100 UmL^−1^ CAT, 200 mM NaCl + 200 UmL^−1^ CAT, and 200 mM NaCl + 300 UmL^−1^ CAT under white light conditions (300 µmol m^−2^ s^−1^) for 8 h. Transverse sections from cladode were observed with a light microscope. Scale bar corresponds to 100 μm.

To determine if the exogenous catalase (CAT) application may counteract the aggregative effect of chloroplasts under salt stress, we incubated cladode segments in solutions with 200 mM NaCl supplemented with 100, 200, and 300 UmL^−1^ CAT during 8 h (Fig. 5). Under the application of 100 UmL^−1^ CAT, we observed that chloroplast grouping was diminished in comparison with the treatment of 200 mM NaCl without CAT. Conversely, we detected that the chloroplasts were completely dispersed throughout the cell when the highest concentrations of CAT were applied (200 and 300 UmL^−1^). In this regard, our data indicate that the application of CAT may inhibit the clustering of chloroplasts during salt stress in *O*. *streptacantha* chlorenchyma cells.

### Catalase alleviated the H_2_O_2_ accumulation caused by salt stress

**Figure 6:**
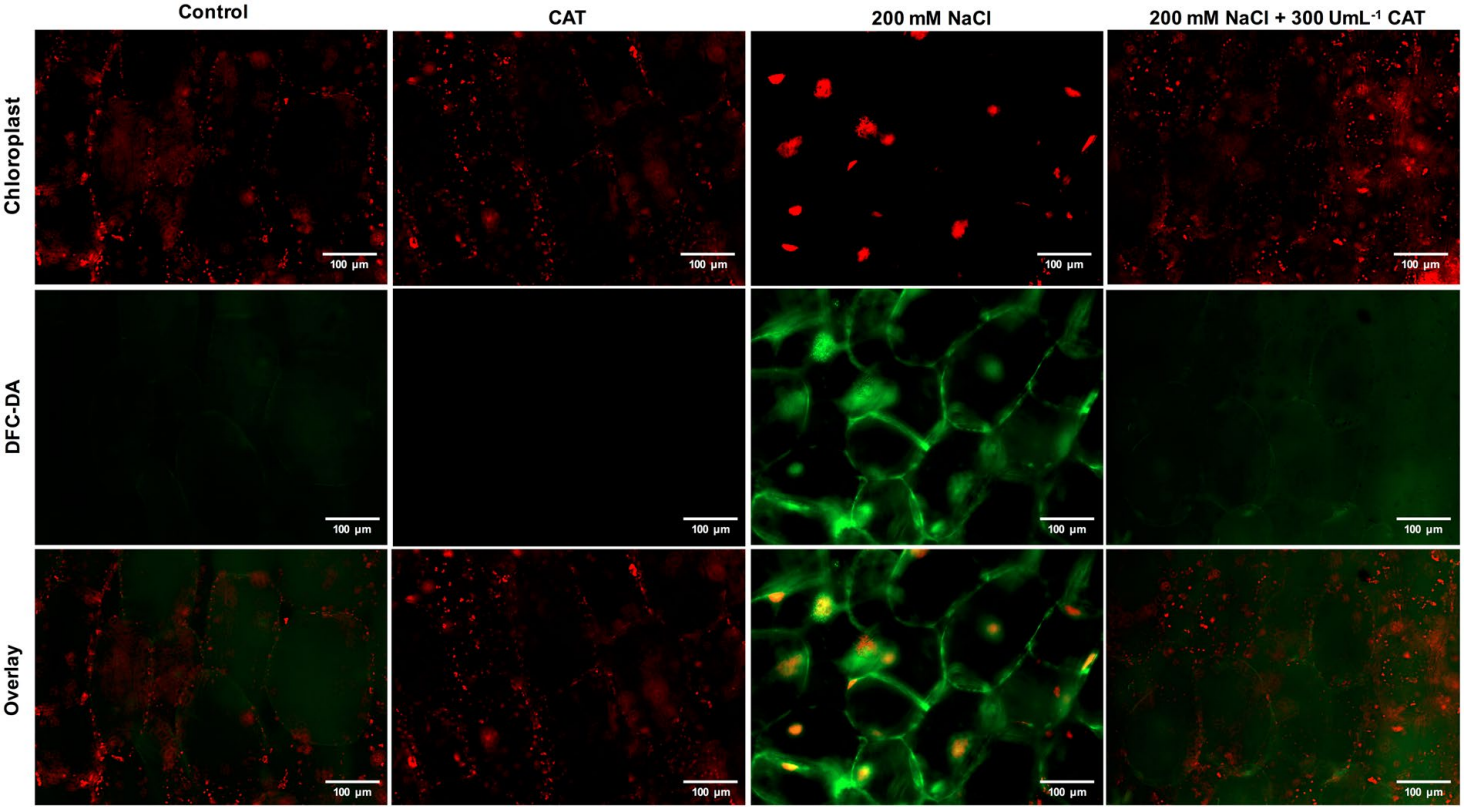
Effects of exogenous CAT application on H_2_O_2_ in the *Opuntia streptacantha* cells exposed to 200 mM NaCl. Representative images of *O*. *streptacantha* cells and their chloroplasts are shown in each panel. Segments of cladode were exposed to 200 mM NaCl, 200 mM NaCl + 300 UmL^−1^ CAT and as control 300 UmL^−1^ CAT under white light conditions (300 µmol m^−2^ s^−1^) for 8 h. Consequently, all treatments were incubated with 25 μM 2′, 7′–dichlorofluorescein diacetate (DCF-DA). Changes of fluorescence intensity in cells was observed using an Epi-fluorescence microscope. Scale bar corresponds to 100 μm.

In order to determine if the exogenous CAT application reduces the oxidative stress by H_2_O_2_ scavenging activity under salt treatment, we monitored the H_2_O_2_ using the fluorescent signals of specific fluorescent probes (DCF-DA) on *O*. *streptacantha* cladode cells exposed to 200 mM NaCl + 300 UmL^−1^ CAT during 8 h (Fig. 6). We found a marked increase of H_2_O_2_ production in cells under 200 mM NaCl compared to the control treatment, whereas, accumulation of H_2_O_2_ was avoided under salt treatment by the application of 300 U mL^−1^ CAT. Thus, our results indicate that the H_2_O_2_ accumulation in *O*. *streptacantha* cells induced by salt stress can be scavenged *in vitro* by CAT activity.

### Catalase confers protection to the photosynthesis process under salt treatment

**Figure 7:**
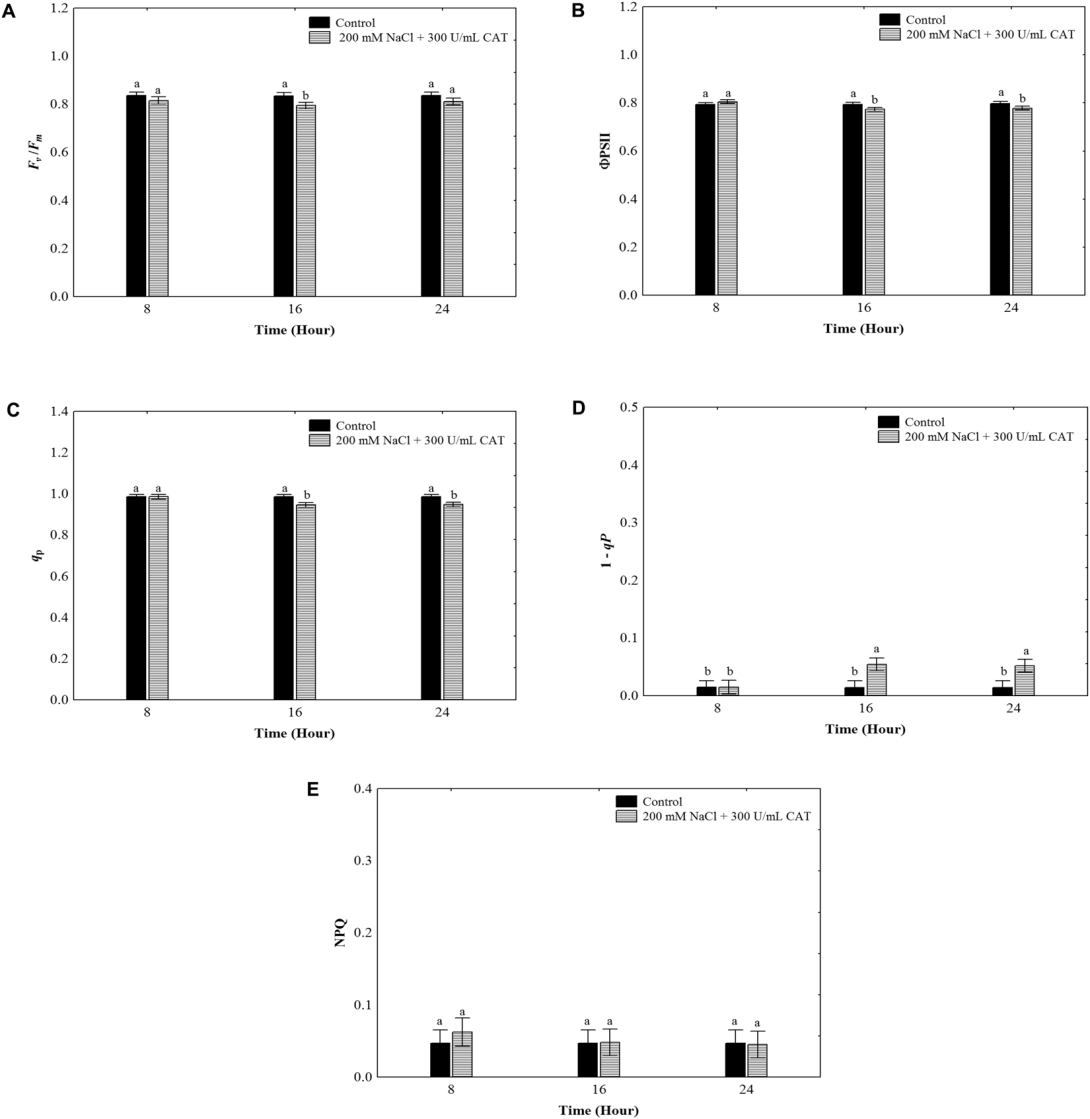
Effects of exogenous CAT application on chlorophyll fluorescence parameters in *Opuntia streptacantha* cladodes exposed to 200 mM NaCl. (**A**) The maximum quantum yield of PSII (*F*_*V*_/*F*_*m*_). (**B**) The effective photochemical quantum yield of PSII (ΦPSII). (**C**) The photochemical quenching (*qP*). (**D**) The excitation pressure (1–*qP*). (**E**) The non-photochemical fluorescence quenching (NPQ). Values are means and bars indicate ± SD, (*n* = 9). Different letters indicate significant difference between treatments and time (hours) according to Duncan’s multiple range tests at *P* < 0.05.

To define if the exogenous CAT application confers protection to the photosynthetic efficiency under salt treatment, we performed chlorophyll fluorescence measurements on *O*. *streptacantha* cladode cells exposed to 200 mM NaCl supplemented with 300 UmL^−1^ CAT during 8, 16, and 24 h. The values of *F*_v_/*F*_*m*_, ϕPSII, *qP*, (1-*qP*), and NPQ remained close to the control without salt stress (Fig. 7A–E), accomplishing that CAT mitigated the negative effects observed at 200 mM NaCl (Fig. 1). In addition, we observed that the CAT application increased the ETR values in cladodes under salt stress (Supplementary Fig. S3) compared to those cladodes treated only with 200 mM NaCl (Supplementary Fig. S1). Our results show that exogenous catalase allows the operation of the photosynthetic machinery without signs of inhibition in *O*. *streptacantha* cladodes under that salt treatment.

### Catalase prevented photosynthetic pigments degradation

To assess whether the exogenous application of CAT prevents the pigments degradation, we quantified the content of chlorophylls and carotenoids in *O*. *streptacantha* cladode segments exposed to 200 mM NaCl supplemented with 300 UmL^−1^ CAT for 8, 16, and 24 h.

**Figure 8:**
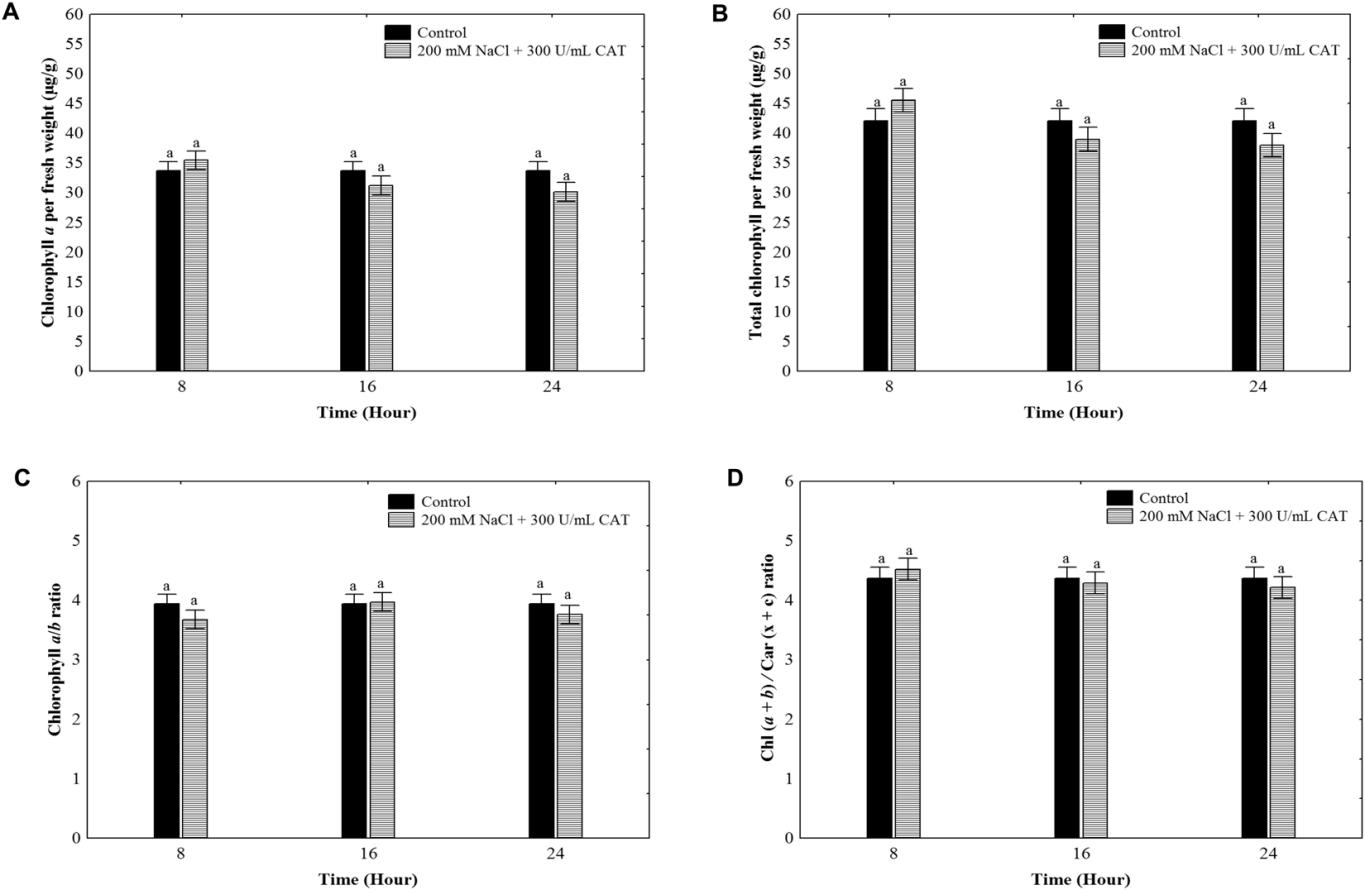
Effects of exogenous CAT application on chlorophyll and carotenoids content in *Opuntia streptacantha* cladodes exposed to 200 mM NaCl. (**A**) Chlorophyll *a*. (**B**) Total chlorophyll (*a* + *b*). (**C**) Chlorophyll *a*/*b* ratio. (**D**) Chl (*a* + *b*)/Car (x + c) ratio. Values are means and bars indicate ± SD, (*n* = 9). Different letters indicate significant difference between treatments and time (hours) according to Duncan’s multiple range tests at *P* < 0.05.

As we previously described in Fig. 2, the treatment of 200 mM NaCl showed a decrement of chlorophyll content. However, CAT exogenous application reversed the negative effect of 200 mM NaCl on chlorophyll levels, reaching values similar to the control without salt stress (Fig. 8). These results support the notion that exogenous CAT prevents photosynthetic pigments degradation induced by salt stress in *O*. *streptacantha* cladodes.

## Discussion

Abiotic stress affects the development, establishment and survival of wild-plants and crops. Photosynthesis is one of the most important physiological processes through which plants produce the essential energy for their growth and development^16^. Chloroplasts are specialized organs that capture sunlight required to perform photosynthesis^1^. However, when plants are exposed to various environmental stresses, such as salinity^10, 17^, drought^18^, or high light conditions^31^, plant’s chloroplasts can get damaged, leading to photosynthesis inhibition.

In this study, we analyzed the effect of salt stress on the movement of chloroplasts in the chlorenchyma cells of *O*. *streptacantha*. Notably, we observed that the chloroplasts were densely clumped towards the center of the cell in response to NaCl treatments. Similar results were reported by Yamada *et al*.^10^, who showed that salinity stress induced aggregative movement of chloroplasts in *Eleusine coracana* cells under normal intensity light. The increase of salt causes adverse effects on the functions and structure of the photosynthetic apparatus, leading to a decline in the *F*_*v*_/*F*_*m*_, ΦPSII, and *qP* parameters. These changes indicate that the reaction centers (RCs) got photochemically inactive, which reduced electron transport capacity in PSII and that decreased the photosynthetic capacity of *O*. *streptacantha* cladodes under salt stress.

In addition, the effect of NaCl treatments on the photosynthetic pigment content in chlorenchyma cells of *O*. *streptacantha* cladodes was analyzed. Our data showed that the decrease in the content of chlorophylls at 8 and 24 h was more pronounced at a low than at a higher salt concentration. One possible explanation could be that *O*. *streptacantha* cells, when sensing high salt concentrations activate several mechanisms to induce *de novo* chlorophyll synthesis, and thus counteract osmotic and ionic shock. This behavior has been previously described in cell lines of *Medicago sativa* and *Nicotiana tabacum*, which showed a greater accumulation of chlorophylls under salt stress^32, 33^. These authors postulate that these changes may be an indicative of physiological processes activation in chloroplasts against saline stress. Therefore, *O*. *streptacantha* cells could activate mechanisms such as chloroplast biogenesis, osmoprotectants synthesis pathways, detoxification, heat shock protein and late embryogenesis abundant proteins involved in chloroplast protection. Moreover, no significant changes in the chlorophyll content at 16 h of NaCl treatments was observed, which could be a feature of acclimation by the re-establishing cellular ion homeostasis in cladode segments under salt stress.

We showed that salt stress causes photosynthesis damage, which in turn would provoke the clustering of chloroplasts like a mechanism of protection to prevent light to penetrate to deeper layers and then reduce the photo-oxidation. However, plants have additional mechanisms to protect themselves against damage by an excess of energy^34^. The reduction in the chlorophyll *a*/*b* ratio in cladode segments under the salt treatments showed in *O*. *streptacantha* can be interpreted as an enlargement in the light-harvesting complex II (LHCII) antenna of PSII. Thus, we suggest that the increase in antenna size could reduce the excitation energy from the antenna to RCs of PSII, or as a direct response of photosynthetic apparatus to salt stress. Similar results were presented by Demetriou *et al*.^35^ who reported that salt stress triggered an increase in the photosynthetic effective antenna size in *Scenedesmus obliquus* under low light conditions.

Moreover, it has been demonstrated that a re-arrangement on antenna leads to the stacking of thylakoid membranes^36^. The ultrastructural analysis in *O*. *streptacantha* chloroplast exhibited that salt treatment induced thylakoid membranes stacking at 8 and 16 h. Dynamics in the stacking of the thylakoid membranes are essential for regulatory processes of the photosynthesis under different abiotic stress conditions^34^. The ability to control the lateral separation of PSI from PSII is considered a functional consequence of grana stacking to minimize the formation of ROS in the PSI through of the decrease of electron transport between photosystems^37, 38^. Thus, our results suggest that changes of thylakoid membranes permit a balance in the excitation energy between the two photosystems^37^. These features can therefore be considered as a response of *O*. *streptacantha* cladodes to salt stress.

Instead, the ratio of chlorophyll to carotenoids at 24 h for the NaCl treatments was low, which indicate damage to the photosynthetic machinery. This result was consistent with the breakdown of chlorophyll caused by chloroplast damage with 200 mM NaCl at 24 h. Several studies have reported that salt stress induce the degradation of photosynthetic pigments in plants by the accumulation of toxic ions and ROS, changing the ultrastructure of the photosynthetic apparatus^16, 39–43^.

Although the chloroplasts could experience damage under salt stress, our results suggest that the clumping of chloroplasts plays an important role to minimize the excitation pressure (*1-qP*) on the photosynthetic machinery in *O*. *streptacantha* cladodes. The aggregative arrangement of chloroplasts in *O*. *streptacantha* seedlings also occurs under drought and high sunlight conditions^9^. Our research group has proposed that chloroplasts move towards the vacuole facilitating malate transportation; thus maintaining the photosynthetic activity under water deficit. The chloroplast-clumping phenomenon is a typical mechanism that has been observed in plants as a response to high solar radiation^11^, salinity^10, 39^, drought^7, 9, 44^, and low temperatures^8^. It has been proposed that the aggregation of chloroplasts may provide protection against photodamage and help to maintain the photosynthetic activity under stressful conditions^10, 44, 45^. Therefore, this phenomenon could be a common adaptive strategy used by plants for their survival under abiotic stress.

We also examined the effect of exogenous catalase (CAT) on chloroplasts clumping of *O*. *streptacantha* cladodes under NaCl treatments. Our data showed that CAT avoided the chloroplast clumping in chlorenchyma cells. Therefore, CAT generated an increase in the *F*_v_/*F*_m_, ΦPSII, *qP* values, and photosynthetic pigment levels in cladodes under salt treatments. Also, the cladode segments treated with CAT experienced less photo-inhibition under salt treatment than those without CAT. These results showed that the CAT application improved the photosynthesis under salt stress, counteracting for the clustering of chloroplasts. Similar photosynthesis protective mechanisms in plants under salt stress have been showed with exogenous application of non-enzymic antioxidants such as ascorbate (AsA)^46, 47^, glutathione (GSH)^48, 49^, and ∝-tocopherols^50^. It is known that enzymatic antioxidants such as CAT provides a very energy-efficient mechanism to remove H_2_O_2_^51^. A high level of endogenous CAT is essential to maintain the antioxidant system that protects plants from oxidative damage due to various environmental stresses^52^. Thus, the exogenous CAT applied to cladode segments of *O*. *streptacantha* produced a protection against oxidative damage by H_2_O_2_ scavenging activity.

The apoplast is an important site for H_2_O_2_ production in acclimation response of plants to salinity^19, 53, 54^. It has been proposed that H_2_O_2_ accumulation in the apoplast could activate a signal for the chloroplast due to their location close to the plasma membrane. Then, the chloroplast may transmit the ROS signaling to the nucleus for the photosynthesis acclimation through nuclear gene expression^20^. Moreover, Wen and Zhang^55^ reported that high blue light exposure can induce H_2_O_2_ generation in the plasma membrane, and that H_2_O_2_ is involved in chloroplast movements in *Arabidopsis thaliana*. Thus, we proposed that H_2_O_2_ generated by salt stress could be acting as a signaling molecule which promotes clumping of chloroplasts, particularly as an acclimation mechanism for mitigation of photo-inhibition in salt-stressed cladodes of *O*. *streptacantha*. However, the role of H_2_O_2_ in chloroplast movement is still poorly understood.

In higher plants, ROS and cytosolic Ca^2+^ ([Ca^2+^]_cyt_), are largely recognized to be important signaling messengers of many biological responses^56^. It is known that high NaCl concentrations, particularly under excess of chloride (Cl^−^) ions in the cytoplasm, leads to an increase of [Ca^2+^]_cyt_ concentrations, which initiates the stress signal transduction pathways in plants under salt stress^57–59^. Furthermore, studies in Arabidopsis leaves reported that calcium is involved in the signal transduction for the movement of chloroplast in response to blue light^5, 60–62^. The authors work shows that the stress by high blue light can increase [Ca^2+^]_cyt_, which may trigger the activity of nicotinamide adenine dinucleotide phosphate-oxidase (NADPH oxidase) to generate H_2_O_2_. In turn; the H_2_O_2_ generated may promote the chloroplast movements. Thus, it is possible that the clustering of chloroplast in cladodes segments can be regulated by internal Ca^2+^ stores, produced by salt stress. However, the cross-talk between Ca^2+^ and H_2_O_2_ in the regulation of chloroplast movements in *O*. *streptacantha* cells is an open question arising from this work that demands future research efforts. Finally, the physiological responses of CAM cacti to salinity are complex and the mechanisms underlying this phenomenon have not been completely elucidated, opening a variety of exciting new questions.

## Conclusions

The chloroplasts are usually more sensitive to salinity than other organelles. Our findings show that salt stress causes photosynthesis damage and accumulation of H_2_O_2_ in the *O*. *streptacantha* cells. We suggest that H_2_O_2_ acts as a messenger molecule for the clustering of chloroplasts. The exogenous application of CAT alleviates salt-induced oxidative stress in *O*. *streptacantha* cladodes most likely through H_2_O_2_ scavenging activity. CAT activity avoids chloroplasts clustering and protects photosynthetic machinery function in salt-stressed *O*. *streptacantha*. In this regard, further studies about the effect of H_2_O_2_ in chloroplast clustering will help to understand the role of this phenomenon during stress.

## Materials and Methods

### Plant material

*Opuntia streptacantha* seeds were collected in 2007 by Delgado-Sánchez *et al*.^9^ from Mexquitic de Carmona municipality San Luis Potosi, Mexico, (22°16′N, 101°07′W at 2,020 m asl). Seeds were sowed in LM-1 substrate (Lambert Professional Peat-Based. Québec, Canada) and watered daily until germination. After one month, the seedlings were transplanted in pots containing peat moss substrate (sunshine Mix # 3, Sungro Horticulture Canada Ltd. Agawam, USA). Pots were maintained under greenhouse conditions at 1059 ± 5.5 μmol m^−2^ s^−1^ photosynthetic photon flux density (PPFD) with 23 °C average air temperature, and watered every other day, during six months. Then, the plants were transferred to a growth chamber for one month at 25 °C under 16 h light (300 μmol m^−2^ s^−1^ PPFD) and 8 h dark cycle.

### NaCl and Catalase treatments

Segments of cladode (5 × 8 × 0.3 mm) were hand-cut in transverse sections from *O*. *streptacantha* plants. For salt treatments, the cladode segments were completely immersed in NaCl solutions (NaCl dissolved in distilled water at final concentrations of 100, 200, and 300 mM) or on distilled water as control for 8, 16, and 24 h. NaCl concentrations were chosen based on previous studies performed in *Opuntia* species^28, 30, 63, 64^. The assays were performed using three different cladodes of *O*. *streptacantha* plants (biological replicates) and we take three segments of cladode per plant (technical replicates) for each treatment, under continuous white light (300 μmol m^−2^ s^−1^) at 25 °C.

For CAT mixed with NaCl solution treatments, the CAT stock solution was prepared by pre-dissolving 1 mg of CAT (Sigma-Aldrich Ref: C1345) in 1 mL 50 mM potassium phosphate buffer pH 7.0. The CAT solutions at final concentrations of 100, 200, and 300 UmL^−1^ respectively, were added to 200 mM NaCl solution. Subsequently, the cladode segments were completely immersed in these mixtures (NaCl + CAT) for 8, 16, and 24 h. As control, we used distilled water without NaCl or CAT. The CAT concentration was selected according to Aroca^65^. The assays were performed using three different cladodes of *O*. *streptacantha* plants (biological replicates) and we take three segments of cladode per plant (technical replicates) for each treatment, under continuous white light (300 μmol m^−2^ s^−1^) at 25 °C.

### Chlorophyll fluorescence measurements

The chlorophyll fluorescence analysis is a powerful technique that allows to obtain detailed information about of the process of photosynthesis in plants^66^ and can also be applied to know how plants respond to abiotic stress factors^67^. Cladode segments of 10 mm diameter were removed from *O*. *streptacantha* plants using forceps and scalp. Subsequently, these were subjected to the aforementioned treatments. Chlorophyll fluorescence was measured using a MINI-PAM II fluorometer (H. Walz, Effeltrich, Germany) following the manufacturer’s instructions. The chlorophyll fluorescence measurements were realized using a pulse the actinic light with an intensity of 820 μmol m^−2^ s^−1^. The maximum quantum yield of photosystem II (*F*_v_/*F*_m_) was determined after dark adaptation of cladodes segments for 30 min. The *F*_*v*_/*F*_*m*_ values were calculated as described by Kitajima and Butler^68^. On the other hand, light-adapted cladodes segments were used to measure the fluorescence parameters as follows: the effective photochemical quantum yield of PSII was calculated using the equation: ΦPSII = (*F*_m_′ − *F*)/*F*_m_′ = Δ*F*/*F*_m_′ presented by Genty *et al*.^69^. The photochemical quenching was calculated as *qP* = (*F*_m_′ − *F*)/(*F*_m_′ − *F*_O_′) and used to determine the fraction of closed (reduced) PSII reaction centers, also known as excitation pressure, and calculated as 1–*qP*. The non-photochemical fluorescence quenching, NPQ = (*F*_m_ − *F*_m_′)/*F*_m_′ was determined according to Bilger and Björkman^70^. The photosynthetic electron transport rate (ETR) was estimated with the following equation ETR = ΦPSII × PPFD × 0.5 × 0.84, where PPFD is photosynthetic photon flux density, the factor 0.5 assumes that photosystems II and I are similarly excited by the irradiance. The factor 0.84 considers that only 84% incident irradiance will be absorbed by the two photosystems^71^. The assays were performed using three different cladodes of *O*. *streptacantha* plants (biological replicates) and we take three segments of cladode per plant (technical replicates) for each treatment (*n* = 9).

### Determination of chlorophylls and carotenoids content

The chlorophyll (*a* and *b*) and carotenoids extraction was performed according to the methodology reported by Lichtenthaler^72, 73^. The cladode chlorenchyma fresh segments were homogenized in 80% acetone and incubated in dark at 4 °C for 5 min. Subsequently, it was centrifuged at 13,000 rpm at 4 °C for 5 min. The chlorophyll and carotenoids contents were measured in 200 μL supernatant using a microplate reader (Epoch 2, Biotek, Winooski, VT, United States of America) at 663 (chlorophyll *a*), 646 (chlorophyll *b*), and 470 (carotenoids) nm wavelengths. The assays were performed using three different cladodes of *O*. *streptacantha* plants (biological replicates) and we take three segments of cladode per plant (technical replicates) for each treatment (*n* = 9). The pigments content, the ratio of chlorophyll *a* (Chl *a*) to chlorophyll *b* (Chl *b*) (Chl *a*/*b*), and Chl *a* and Chl *b* to total carotenoids (*a* + *b*)/(x + c) ratio were estimated using the equations proposed by Lichtenthaler^73^:

$$\begin{array}{rcl}Chl\,a & = & (12.25\times {A}_{663})-(2.79\times {A}_{646})\\ Chl\,b & = & (21.50\times {A}_{646})-(5.10\times {A}_{663})\\ Chl\,a+Chl\,b & = & (7.15\times {A}_{663})+(18.71\times {A}_{646})\\ Carotenoids\, & = & \frac{(1000\times {A}_{470})-(1.82\times chl\,a)-(85.02\times chl\,b)}{198}\end{array}$$

### Microscope analyses

To analyze the chloroplast arrangement on *O*. *streptacantha* cells, segments of cladode (5 × 8 × 0.3 mm) subjected to the treatments described below were observed without fixation under a light microscope Leica DM2000 (Leica, Wetzlar, Germany). The photographs were obtained and digitized with LAS Imaging Software (Leica). For the transmission electron microscope (TEM), the segments of cladodes were treated with a fixative solution (10% glutaraldehyde in 0.1 M sodium phosphate, pH 7.4) during overnight at 4 °C. Then, they were washed using Sorensen’s phosphate buffer and dehydrated with ethanol; subsequently, the samples were polymerized in a fresh resin at 60 °C as described by Cocoletzi *et al*.^74^. Ultrathin sections were contrasted using aqueous uranyl acetate (2% w/v) and aqueous lead citrate (2% w/v). Samples were examined with TEM JEOL 200CX (JEOL, Welwyn Garden City, UK) using a 100 kV acceleration voltage.

The ultrastructural analysis of chloroplasts to determine the thickness of the thylakoid lumen was realized using the methodology reported by Kirchhoff *et al*.^38^. The distances of stacking repeat unit (*R*), which includes the two thylakoid membrane bilayers (*M*) and the widths of one partition gap (*P*) were realized with Image J software^75^. Subsequent, the thickness of the thylakoid lumen (*L*) were estimated using the equations proposed by Kirchhoff *et al*.^38^: *L* = *R* − *M* − *P*. The measurements of lumen thickness were made on grana lamellae (GL) using three separate micrographs and we take three grana lamellae per micrographs for each treatment (*n* = 9).

### Epi-fluorescence microscope for H_2_O_2_ images

For observer H_2_O_2_ signals, segments of cladode (5 × 8 × 0.3 mm) subjected to the treatments described below were transferred to 25 μM 2′, 7′–dichlorofluorescein diacetate (DCF-DA) (Sigma-Aldrich Ref: C1345) dissolved in 10 mM Tris-HCl, 50 mM KCl (pH 7.2) and incubated in dark for 30 min at 30 °C. Subsequent, the segments were washed twice with 10 mM Tris-HCl, 50 mM KCl (pH 6.1) for 30 min, images were visualized using an Epi-fluorescence microscope (Axio Imager M2; Carl Zeiss Microscopy, LLC, USA). The assays were performed using three different cladodes of *O*. *streptacantha* plants (biological replicates) and we take three segments of cladode per plant (technical replicates) for each treatment (*n* = 9).

### Data analysis

All data obtained from chlorophyll fluorescence parameters and pigments content were statistically analyzed with STATISTICA version 7 software^76^, using Duncan’s multiple range test at the *P* < 0.05 level of significance between treatments and time (hours). The results are expressed as mean values ± SD (standard deviation) (*n* = 9).

## Electronic supplementary material

Supplementary Information

## References

[1] Lambers, H. *et al*. *Plant Physiological Ecology* (Springer Science, 2008).

[2] Van Gestel K, Kohler RH, Verbelen JP (2002). Plant mitochondria move on F-actin, but their positioning in the cortical cytoplasm depends on both F-actin and microtubules. J. Exp. Bot..

[3] Mano S (2002). Distribution and characterization of peroxisomes in Arabidopsis by visualization with GFP: dynamic morphology and actin-dependent movement. Plant Cell Physiol..

[4] Oikawa K (2008). Chloroplast outer envelope protein CHUP1 is essential for chloroplast anchorage to the plasma membrane and chloroplast movement. Plant Physiol..

[5] Wada M (2013). Chloroplast movement. Plant Sci..

[6] Kasahara M, Kagawa T, Oikawa K (2002). Chloroplast avoidance movement reduces photodamage in plants. Nature.

[7] Kondo A, Kaikawa J, Funaguma T, Ueno O (2004). Clumping and dispersal of chloroplasts in succulent plants. Planta.

[8] Tanaka A (2007). Photosynthetic activity in winter needles of the evergreen tree *Taxus cuspidata* at low temperatures. Tree Physiol..

[9] Delgado-Sánchez, P., Yáñez-Espinosa, L., Jiménez-Bremont, J. F., Chapa-Vargas, L. & Flores, J. Ecophysiological and anatomical mechanisms behind the nurse effect: Which are more important? A multivariate approach for cactus seedlings. *PLoS One* **8** (2013). 10.1371/journal.pone.0081513 PMC3842958 24312310

[10] Yamada, M., Kawasaki, M., Sugiyama, T., Miyake, H. & Taniguchi, M. Differential positioning of C_4_ mesophyll and bundle sheath chloroplasts: Aggregative movement of C_4_ mesophyll chloroplasts in response to environmental stresses. *Plant Cell Physiol*., doi:10.1093/pcp/pcp116 (2009). 10.1093/pcp/pcp116 19667101

[11] Maai E (2011). The avoidance and aggregative movements of mesophyll chloroplasts in C_4_ monocots in response to blue light and abscisic acid. J. Exp. Bot..

[12] Lüttge U (2004). Ecophysiology of Crassulacean Acid Metabolism (CAM). Ann. Bot..

[13] Lüttge. Ability of crassulacean acid metabolism plants to overcome interacting stresses in tropical environments. *AoB Plants* (2010). 10.1093/aobpla/plq005 PMC3000696 22476063

[14] Nobel, P. S. *Environmental Biology of Agaves and Cacti*. (Cambridge University Press, 1988).

[15] Goldstein G, Nobel PS (1994). Water relations and low-temperature acclimation for cactus species varying in freezing tolerance. Plant Physiol..

[16] Ashraf M, Harris PJC (2013). Photosynthesis under stressful environments: An overview. Photosynthetica.

[17] Sudhir, P. & Murthy, S. D. Effects of salt stress on basic processes of photosynthesis. **42**, 481–486 (2004).

[18] Chaves MM, Flexas J, Pinheiro C (2009). Photosynthesis under drought and salt stress: Regulation mechanisms from whole plant to cell. Ann. Bot..

[19] Miller G, Suzuki N, Ciftci-Yilmaz S, Mittler R (2010). Reactive oxygen species homeostasis and signalling during drought and salinity stresses. Plant, Cell Environ..

[20] Shapiguzov A, Vainonen JP, Wrzaczek M, Kangasjärvi J (2012). ROS-talk-how the apoplast, the chloroplast, and the nucleus get the message through. Front. Plant Sci..

[21] Shuvasish C, Panda P, Sahoo L & Panda, S. K. Reactive oxygen species signaling in plants under abiotic stress. *Plant Signal*. *Behav*. **8** (2013). 10.4161/psb.23681 PMC7030282 23425848

[22] Foyer CH, Shigeoka S (2011). Understanding oxidative stress and antioxidant functions to enhance photosynthesis. Plant Physiol..

[23] Das K, Roychoudhury A (2014). Reactive oxygen species (ROS) and response of antioxidants as ROS-scavengers during environmental stress in plants. Front. Environ. Sci..

[24] Sharma P, Jha AB, Dubey RS, Pessarakli M (2012). Reactive oxygen species, oxidative damage, and antioxidative defense mechanism in plants under stressful conditions. J. Bot..

[25] Sofo A, Scopa A, Nuzzaci M, Vitti A (2015). Ascorbate peroxidase and catalase activities and their genetic regulation in plants subjected to drought and salinity stresses. Int. J. Mol. Sci..

[26] Foyer CH, Noctor G (2005). Redox homeostasis and antioxidant signaling: a metabolic interface between stress perception and physiological responses. Plant Cell.

[27] Logan BA, Kornyeyev D, Hardison J, Holaday AS (2006). The role of antioxidant enzymes in photoprotection. Photosynth. Res..

[28] Nerd A, Karadi A, Mizrahi Y (1991). Salt tolerance of prickly pear cactus (*Opuntia ficus-indica*). Plant Soil.

[29] Murillo-Amador B, Cortes-Avila A, Troyo-Dieguez E, Nieto-Garibay A, Jones H (2001). Efects of NaCl salinity on growth and production of young cladodes of *Opuntia Ficus-indica*. J. Agron. Crop Sci..

[30] Silva-Ortega CO, Ochoa-Alfaro AE, Reyes-Agüero JA, Aguado-Santacruz GA, Jiménez-Bremont JF (2008). Salt stress increases the expression of *p5cs* gene and induces proline accumulation in cactus pear. Plant Physiol. Biochem..

[31] Li Z, Wakao S, Fischer BB, Niyogi KK (2009). Sensing and responding to excess light. Annu. Rev. Plant Biol..

[32] Winicov I, Button JD (1991). Accumulation of photosynthesis gene transcripts in response to sodium chloride by salt-tolerant alfalfa cells. Planta.

[33] Locy RD, Chang CC, Nielsen BL, Singh NK (1996). Photosynthesis in salt-adapted heterotrophic tobacco cells and regenerated plants. Plant Physiol..

[34] Kirchhoff H (2013). Architectural switches in plant thylakoid membranes. Photosynth. Res..

[35] Demetriou G, Neonaki C, Navakoudis E, Kotzabasis K (2007). Salt stress impact on the molecular structure and function of the photosynthetic apparatus-The protective role of polyamines. Biochim. Biophys. Acta - Bioenerg..

[36] Kaňa, R. & Govindjee Role of ions in the regulation of light-harvesting. *Front*. *Plant Sci*. **7** (2016). 10.3389/fpls.2016.01849 PMC5160696 28018387

[37] Chow WS, Kim E-H, Horton P, Anderson JM (2005). Granal stacking of thylakoid membranes in higher plant chloroplasts: the physicochemical forces at work and the functional consequences that ensue. Photochem. Photobiol. Sci..

[38] Kirchhoff H (2011). Dynamic control of protein diffusion within the granal thylakoid lumen. Proc. Natl. Acad. Sci..

[39] Gao H-J (2015). Ultrastructural and physiological responses of potato (*Solanum tuberosum* L.) plantlets to gradient saline stress. Front. Plant Sci..

[40] Shu S, Yuan L, Guo S, Sun J, Yuan Y (2013). Effects of exogenous spermine on chlorophyll fluorescence, antioxidant system and ultrastructure of chloroplasts in *Cucumis sativus* L. under salt stress. Plant Physiol. Biochem..

[41] Mitsuya S, Takeoka Y, Miyake H (2000). Effects of sodium chloride on foliar ultrastructure of sweet potato (*Ipomoea batatas* Lam.) plantlets grown under light and dark conditions *in vitro*. J. Plant Physiol..

[42] Yamane K, Rahman MS, Kawasaki M, Taniguchi M, Miyake H (2004). Pretreatment with antioxidants decreases the effects of salt stress on chloroplast ultrastructure in rice leaf segments (*Oryza sativa* L.). Plant Prod. Sci..

[43] Oyiga BC (2016). Identification and characterization of salt tolerance of wheat germplasm using a multivariable screening approach. J. Agron. Crop Sci..

[44] Maai E, Miyake H, Taniguchi M (2011). Differential positioning of chloroplasts in C_4_ mesophyll and bundle sheath cells. Plant Signal. Behav..

[45] Murchie EH, Chen Y, Hubbart S, Peng S, Horton P (1999). Interactions between senescence and leaf orientation determine *in situ* patterns of photosynthesis and photoinhibition in field-grown rice. Plant Physiol..

[46] Shalata A, Neumann PM (2001). Exogenous ascorbic acid (vitamin C) increases resistance to salt stress and reduces lipid peroxidation. J Exp Bot..

[47] Athar H (2008). ur R., Khan, A. & Ashraf, M. Exogenously applied ascorbic acid alleviates salt-induced oxidative stress in wheat. Environ. Exp. Bot..

[48] Rawia AE, Taha LS, Ibrahiem SMM (2011). Alleviation of adverse effects of salinity on growth, and chemical constituents of marigold plants by using glutathione and ascorbate. J. Appl. Sci. Res..

[49] Ahmad, P., Azooz, M. M. & Prasad, M. N. V. *Ecophysiology and responses of plants under salt stress* (Springer Science, 2013).

[50] Farouk S (2011). Ascorbic acid and α-Tocopherol minimize salt-induced wheat leaf senescence. J. Stress Physiol. Biochem..

[51] Scandalios JG (2005). Oxidative stress: Molecular perception and transduction of signals triggering antioxidant gene defenses. Brazilian J. Med. Biol. Res..

[52] Shigeoka S (2002). Regulation and function of ascorbate peroxidase isoenzymes. J. Exp. Bot..

[53] Neil S, Desekan R, Hancock J (2002). Hydrogen peroxide signalling. Curr. Opin. Plant Biol..

[54] Slesak I, Libik M, Karpinska B, Karpinski S, Miszalski Z (2007). The role of hydrogen peroxide in regulation of plant metabolism and cellular signalling in response to environmental stresses. Acta Biochim Pol.

[55] Wen F, Xing D, Zhang L (2008). Hydrogen peroxide is involved in high blue light-induced chloroplast avoidance movements in *Arabidopsis*. J. Exp. Bot..

[56] Mazars C, Thuleau P, Lamotte O, Bourque S (2010). Cross-talk between ROS and calcium in regulation of nuclear activities. Mol. Plant.

[57] Tavakkoli E, Fatehi F, Coventry S, Rengasamy P, McDonald GK (2011). Additive effects of Na^+^ and Cl^−^ ions on barley growth under salinity stress. J. Exp. Bot..

[58] Tuteja N, Sopory SK (2008). Chemical signaling under abiotic stress environment in plants. Plant Signal. Behav..

[59] Kader MA, Lindberg S (2010). Cytosolic calcium and pH signaling in plants under salinity stress. Plant Signal. Behav..

[60] Suetsugu N, Wada M (2007). Chloroplast photorelocation movement mediated by phototropin family proteins in green plants. Biol. Chem..

[61] Kong S-G (2013). Both phototropin 1 and 2 localize on the chloroplast outer membrane with distinct localization activity. Plant Cell Physiol..

[62] Labuz J (2016). Blue light-dependent changes in loosely bound calcium in *Arabidopsis* mesophyll cells: An X-ray microanalysis study. J. Exp. Bot..

[63] Nobel PS, Lurjge U, Heuer S, Ball E (1984). Influence of applied NaCl on Crassulacean Acid Metabolism and ionic levels in a cactus, *Cereus validus*. Plant Physiol..

[64] Silverman FP, Young DR, Nobel PS (1988). Effects of applied NaCl on *Opuntia humifusa*. Physiol. Plant..

[65] Aroca R (2006). Exogenous catalase and ascorbate modify the effects of abscisic acid (ABA) on root hydraulic properties in *Phaseolus vulgaris* L. plants. J. Plant Growth Regul..

[66] Maxwell K, Jonhson GN (2000). Chlorophyll fluorescence - a practical guide. J. Exp. Bot..

[67] Murchie EH, Lawson T (2013). Chlorophyll fluorescence analysis: A guide to good practice and understanding some new applications. J. Exp. Bot..

[68] Kitajima M, Butler WL (1975). Quenching of chlorophyll fluorescence and primary photochemistry in chloroplasts by dibromothymoquinone. Biochim. Biophys. Acta - Bioenerg..

[69] Genty B, Briantais JM, Baker NR (1989). The relationship between the quantum yield of photosynthetic electron transport and quenching of chlorphyll fluorescence. Biochim. Biophys. Acta.

[70] Bilger W, Björkman O (1990). Role of the xanthophyll cycle in photoprotection elucidated by measurements of light-induced absorbance changes, fluorescence and photosynthesis in leaves of *Hedera canariensis*. Photosynth. Res..

[71] Rascher U, Liebig M, Lüttge U (2000). Evaluation of instant light-response curves of chlorophyll fluorescence parameters obtained with a portable chlorophyll fluorometer on site in the field. Plant, Cell Environ..

[72] Lichtenthaler, H. & Buschamann, C. Extraction of photosynthetic tissues: chlorophylls and carotenoids. *Curr*. *Protoc*. *Food Anal*. *Chem*. (2001).

[73] Lichtenthaler H (1987). Chlorophylls and carotenoids: pigments of photosynthetic biomembranes. Methods Enzymol..

[74] Cocoletzi E, Guillermo A, Gregório C, Araceli P, Juan Francisco O (2016). Bidirectional anatomical eff ects in a mistletoe–host relationship: *Psittacanthus schiedeanus* mistletoe and its hosts *Liquidambar styraciflua* and *Quercus germana*. Am. J. Bot..

[75] Abràmoff MD, Magalhães PJ, Ram SJ (2005). Image processing with ImageJ Part II. Biophotonics Int..

[76] StatSoft, I. SS I., ‘Statistica’, Data analysis software system, version 7. (2004).

