# Supplementary material for: Effects of catalase on chloroplast arrangement in Opuntia streptacantha chlorenchyma cells under salt stress
Source: Sci Rep. 2017 Aug 17;7:8656. doi: 10.1038/s41598-017-08744-x (PMC5561099; doi:10.1038/s41598-017-08744-x)
Supplement: Supplementary file 1 — Supplementary Information [file 41598_2017_8744_MOESM1_ESM.pdf]

Supplementary information

Effects of catalase on chloroplast arrangement in *Opuntia streptacantha* chlorenchyma cells under salt stress

Diana Marcela Arias-Moreno[1], Juan Francisco Jiménez-Bremont[2], Israel Maruri-López[2], Pablo Delgado-Sánchez[1*]

[1]Laboratorio de Biotecnología, Facultad de Agronomía y Veterinaria, Universidad Autónoma de San Luis Potosí, Soledad de Graciano Sánchez, SLP., México.

[2]Laboratorio de Biología Molecular de Hongos y Plantas, División de Biología Molecular, Instituto Potosino de Investigación Científica y Tecnológica A.C, San Luis Potosí, SLP, México.

All authors contributed equally to this work. *Correspondence and requests for materials should be addressed to Pablo Delgado-Sánchez (email: pablo.delgado@uaslp.mx).

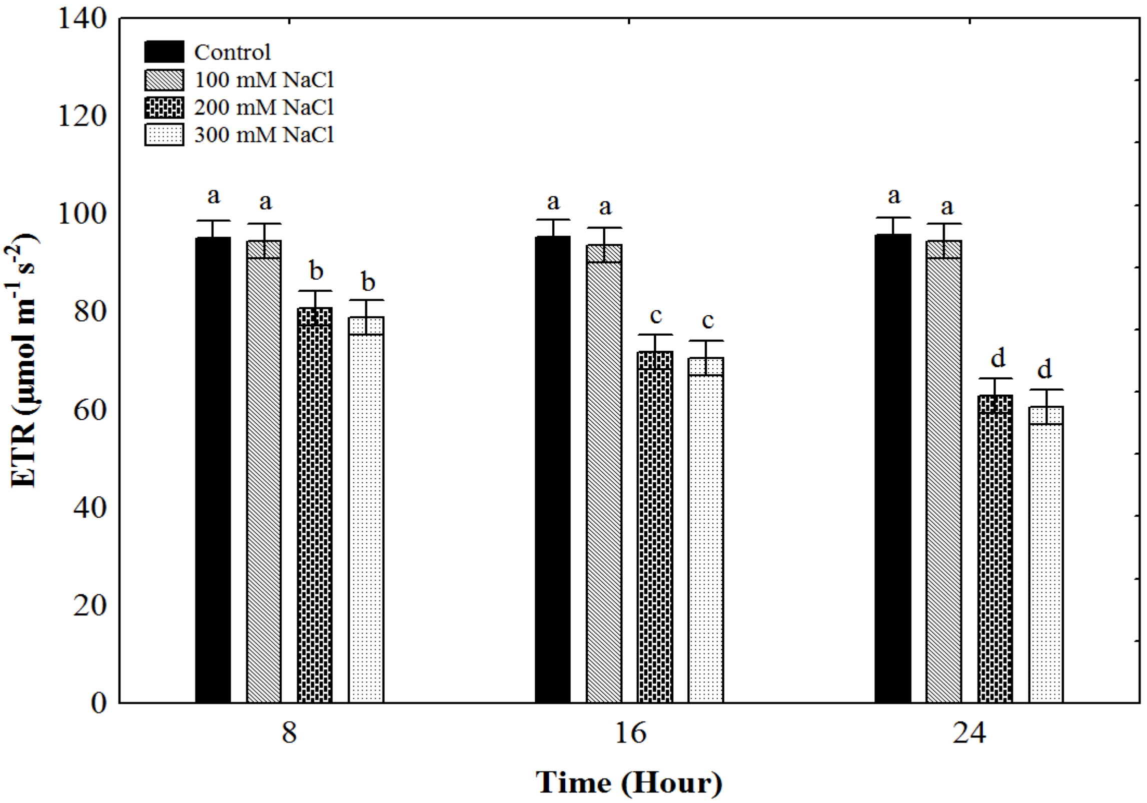

Figure S1. The photosynthetic electron transport rate (ETR) in *Opuntia streptacantha* cladodes under salt treatments. Segments of cladode were exposed to 100, 200, and 300 mM NaCl under white light conditions (300 µmol m^{-2} s^{-1}) for 8, 16, and 24 h. (100, 200, and 300 mM NaCl), during 8, 16, and 24 h. Values are means and bars indicate ± SD, (*n*=9). Different letters indicate significant difference between treatments and time (hours) according to Duncan's multiple range tests at $P < 0.05$.

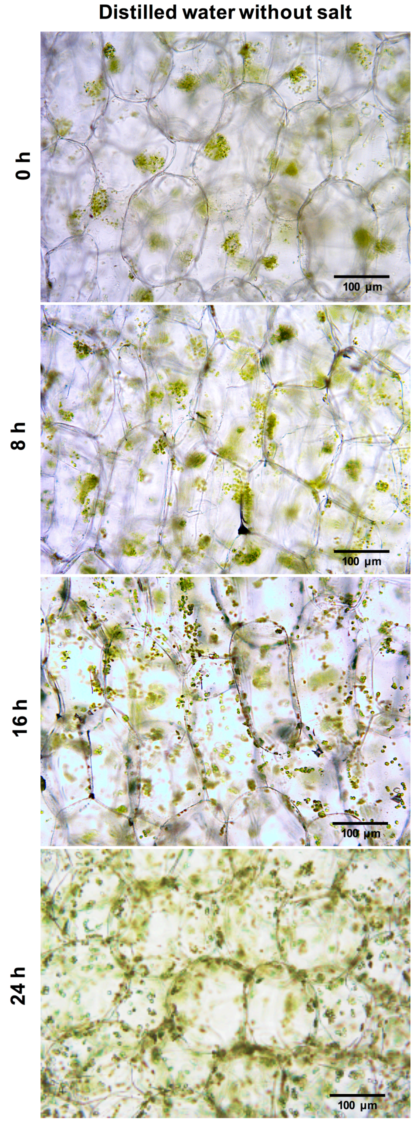

Figure S2. Chloroplast arrangement in salt stressed cladode segments incubated in distilled water without salt. Representative images of *Opuntia streptacantha* cells and their chloroplast are shown in each panel. Cladode segments of *O. streptacantha* plants were incubated in 200 mM NaCl for 8 h. Afterwards, they were washed and incubated in distilled water without NaCl under continuous white light (300 µmol m^{-2} s^{-1}) for 0, 8, 16, and 24 h. Scale bar corresponds to 100 µm.

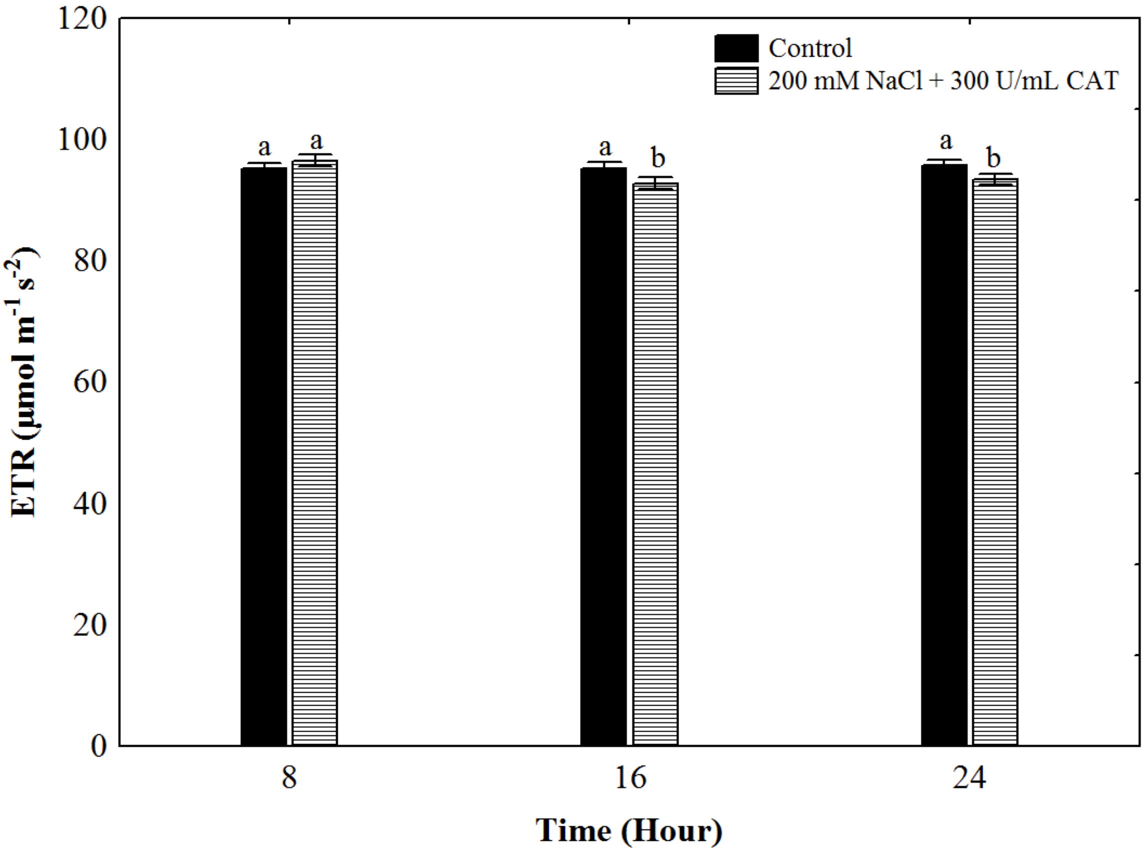

Figure S3. The photosynthetic electron transport rate (ETR) in *Opuntia streptacantha* cladodes under NaCl treatment supplemented with catalase. Segments of cladode were exposed to 200 mM NaCl and 200 mM NaCl + 300 UmL^{-1} CAT under white light conditions (300 µmol m^{-2} s^{-1}) for 8, 16, and 24 h. Values are means and bars indicate ± SD, (*n*=9). Different letters indicate significant difference between treatments and time (hours) according to Duncan's multiple range tests at $P < 0.05$.
